# Clinical and Cardiovascular Magnetic Resonance Predictors of Early and Long-Term Clinical Outcome in Acute Myocarditis

**DOI:** 10.3389/fcvm.2022.886607

**Published:** 2022-04-29

**Authors:** Yohann Bohbot, Jérôme Garot, Thomas Hovasse, Thierry Unterseeh, Chloé Di Lena, Wahiba Boukefoussa, Chloé Tawa, Cédric Renard, Isabelle Limouzineau, Suzanne Duhamel, Philippe Garot, Christophe Tribouilloy, Francesca Sanguineti

**Affiliations:** ^1^Department of Cardiology, Amiens University Hospital, Amiens, France; ^2^Institut Cardiovasculaire Paris Sud (ICPS), CMR Department, Hôpital Privé Jacques Cartier, Ramsay Santé, Massy, France; ^3^Department of Radiology, Amiens University Hospital, Amiens, France

**Keywords:** cardiovascular magnetic resonance, outcome, late gadolinium enhancement, left ventricular ejection fraction, myocarditis

## Abstract

**Introduction:**

The natural history of acute myocarditis (AM) remains partially unknown and predictors of outcome are debated. We sought to assess the impact of various cardiac magnetic resonance (CMR) parameters on early and long-term prognosis in a population of patients with AM.

**Materials and Methods:**

In a two-center longitudinal study, we included consecutive patients with diagnosis of AM based on CMR and without hemodynamic compromise. The primary endpoint was the occurrence of an event in the acute phase (≤15 days). Secondary endpoints were the occurrence of major adverse cardiac events (MACE) and recurrence of AM during follow-up.

**Results:**

Three hundred and eighty-eight patients were included [mean age 38.5 years, 77.3% male, mean ejection fraction (EF):56%] of which 82% (317) presented with chest pain. CMR was performed 4 ± 2 days after index presentation. Overall, 38 patients (9.8%) had an event at the acute phase, 41 (10.6%) presented at least one MACE during follow-up (median 7.5 years, 6.6–8.9) and 30 (7.7%) experienced a recurrence of AM. By multivariate analysis, the independent predictors of initial complications were absence of chest pain (OR [95%CI] = 0.35 [0.15–0.82]), presence of syncope/pre-syncope (OR [95%CI] = 3.56 [1.26–10.02]), lower EF (OR [95%CI] = 0.94 [0.91–0.98] per%), myocardial extent of late gadolinium enhancement (LGE) (OR [95%CI] = 1.05 [1.002–1.100] per%) and absence of edema (OR [95%CI] = 0.44 [0.19–0.97]). Only age (HR [95%CI] = 1.021 [1.001–1.041] per year) and an initial alteration of EF (HR [95%CI] = 0.94 [0.91–0.97] per%) were associated with MACE during follow-up. Factors independently associated with AM recurrence were myocarditis prior to the index episodes (HR [95%CI] = 5.74 [1.72–19.22]) and viral syndrome at the index episode (HR [95%CI] = 4.21 [1.91–9.28]).

**Conclusion:**

In routine consecutive hemodynamically stable patients with diagnosis of AM based on CMR, absence of edema, reduced EF, and extent of LGE were associated with early adverse outcome. Only age and EF were associated with long-term events.

## Introduction

Acute myocarditis (AM) has an underlying viral etiology in most cases and remains difficult to diagnose because of heterogeneous clinical presentations (1). Although the vast majority of patients have no hemodynamic compromise at the acute stage, AM may have a fulminant presentation. It may also lead to potentially life-threatening complications and subsequently to dilated cardiomyopathy and heart failure (2). Endomyocardial biopsy (EMB) is the current gold standard to confirm the diagnosis of AM, but it is invasive and may lack sensitivity due to the focal nature of the disease (3–5). For these reasons, its use is currently limited to severe forms of AM. Indeed, its main interest is to identify particular etiologies that could benefit from specific treatments (3, 5). Cardiovascular magnetic resonance (CMR) has been recognized as the most appropriate non-invasive diagnostic method for AM diagnosis (6, 7). Besides the evaluation of left ventricular (LV) volumes and function, it carries unprecedented diagnostic value for the accurate depiction of myocardial inflammatory lesions and irreversible myocardial damage (6–13). Some CMR parameters have been identified as potential predictors of outcome, although their value remains controversial (14). Previous studies showed that the presence, extent and location of late gadolinium enhancement (LGE) at the acute phase were predictive of poor prognosis (15–17), whereas others did not (18, 19). Those apparent discrepancies may be due to relatively short clinical follow-up in most studies, and especially to the fact that the patient populations are heterogeneous. The study sought to assess the impact of various CMR parameters on early and long-term prognosis in a population of patients with AM diagnosed by CMR.

## Materials and Methods

### Study Protocol

This retrospective study was conducted in two French tertiary centers [Institut Cardiovasculaire Paris Sud (ICPS) and Amiens University hospital] and included consecutive patients with a CMR-based diagnostic of AM. Patients from ICPS (*n* = 203) were included between October 2008 and December 2011 as previously described (18) and patients from Amiens University hospital (*n* = 185) were included between 2010 and 2015. Patients who underwent CMR at the time of acute presentation with at least 3 of the following criteria: (1) chest pain, (2) a recent episode (< 1 month) of acute viral infection, (3) repolarization abnormalities on electrocardiogram, (4) elevated troponin, were eligible for this study. A coronary angiography was performed in case of significant ST segment elevation on the ECG or at the discretion of the physicians when deemed appropriate. The diagnosis of AM was based on the presence of intramyocardial and/or subepicardial LGE indicative of myocardial damage, and at least 1 of the following criteria: (1) presence of myocardial edema identified by the presence of spontaneous subepicardial and/or intramyocardial increased signal intensity on T2-weighted spin echo images, (2) subepicardial and/or intramyocardial early gadolinium enhancement indicative of hyperemia, and (3) absence of microvascular obstruction and subendocardial LGE. Exclusion criteria were: (1) Age < 18 years, (2) the presence of severe life-threatening non-cardiac disease, (3) presence of other cardiac disease, and (4) severe hemodynamic compromise that precluded the CMR study. When AM was diagnosed by CMR, medical treatment usually included β-blockers and angiotensin-converting enzyme inhibitors for at least 6 weeks and aspirin if there was an associated pericarditis. In case of acute or subacute heart failure or rhythm disorders, specific treatments were given when appropriate. The study was approved by both institutions local Ethic Committees and conducted in compliance with institutional policies, national legal requirements and the revised principles of the Declaration of Helsinki. The data underlying this article will be shared on reasonable request to the corresponding author.

### Cardiovascular Magnetic Resonance Acquisitions

CMR was performed between within 7 days of first symptoms using a Siemens Magnetom Espree^®^ 1.5 T scanner (Erlangen, Germany) and a General Electric Optima MR450 W, 1.5 T (Milwaukee, Wisconsin, United States) using an 8-element phased-array coil. Cine-MR images in the long axis (2, 3, and 4 chambers) and in the short axis, covering the left ventricle from the base to the apex, were obtained using a fast-imaging Steady State Free Precession (SSFP) sequence. Myocardial edema was studied in matched locations using a triple inversion-recovery T2-weighted turbo spin echo sequence with fat and blood suppression inversion pulses (T2 STIR). Next, a bolus of gadolinium contrast (0.1 mmol/kg) was injected with an injector at a dose of 4 ml/s. Presence of LGE was assessed 10 min after the administration of the contrast in matched locations through the use of inversion-recovery 2D fast spoiled gradient echo sequence with an inversion time (TI) set to null normal myocardial signal (determined by TI scout sequence).

### Cardiovascular Magnetic Resonance Analysis

All CMR images were independently reviewed by two experienced physicians and, in case of discrepancies, by a third observer to reach a consensus. The myocardium was divided into 17 segments according to the North American Society of Myocardial Imaging consensus (20). LV volumes and ejection fraction were derived by summation of epicardial and endocardial contours. Myocardial edema was defined as a spontaneous hypersignal on black-blood T2-STIR images. LGE extent was evaluated using a semi-quantitative analysis. Each of the 17 myocardial segments of the left ventricle was divided into 3 layers (outer, middle and inner). Myocardial lesions were evaluated in the 3 layers of each segment of the myocardium (18). Myocardial lesions were finally evaluated by planimetry with the use of an automated cut-off greater than 5 *SD* above the mean signal intensity of the myocardium and expressed as a percentage of the total LV myocardial area.

### Follow-Up and Endpoints

The primary clinical endpoint was the occurrence of an event in the acute phase of AM (within 15 days of diagnosis). This criterion was composite and included cardiac death, development or worsening of heart failure (determined by clinicians in each center on the basis of clinical symptoms, signs, laboratory results, and chest X-rays according to the Framingham criteria), sustained ventricular arrhythmia or complete atrioventricular block. Patients were followed over time [median follow-up: 7.5(IQR:6.6–8.9) years] and the secondary clinical endpoints were the occurrence of major adverse cardiac events (MACE) and the recurrence of AM. Events were ascertained by direct patient interview and clinical examination and/or repeated telephone calls to physicians, patients, and (if necessary) next of kin. The adjudication of clinical event was based on a consensus reached by 2 senior cardiologists. MACEs were defined by the occurrence of at least one of the following events: cardiac death, cardiac transplantation, documented sustained ventricular arrythmia, hospitalization for heart failure and hospitalization for cardiac causes. Recurrence of AM was defined as a new episode of clinically suspected AM, at least 6 months after the index episode, with the presence of edema in T2 STIR and LGE.

### Statistical Analysis

Baseline continuous variables are expressed as mean ± standard deviation or median [interquartile range], and categorical variables are expressed as numbers and percentages. The normality of data was assessed using the Skewness and Kurtosis normality test. The differences between the groups were assessed with the chi-square test or Fisher exact test for categorical variables and Student’s *t*-test for continuous variables. Factors associated with the occurrence of events in the acute phase were investigated using multivariate logistic regression analyses including all significant variables in univariate analysis with a *p*-value < 0.1 to ensure that we do not drop any potentially useful variables from the multivariate analysis. As the presence of a LGE was mandatory to retain the diagnosis of AM in this study, we could not test its impact on the prognosis Factors associated with the occurrence of MACE during follow-up were identified using a Cox multivariate analysis that included all variables with a *p*-value < 0.10 in univariate analysis. Colinearity was considered in the multivariate analysis if the Pearson coefficient of correlation was > 0.6 or if a covariate’s standard error was > 5.0. Collinear variables were removed from the multivariate analysis. The limit of statistical significance was *p* < 0.05 and all tests were two-tailed. Data were analyzed using SPSS 27.0 (SPSS Inc. Chicago, IL).

## Results

### Baseline Clinical Characteristics

**FIGURE 1:**
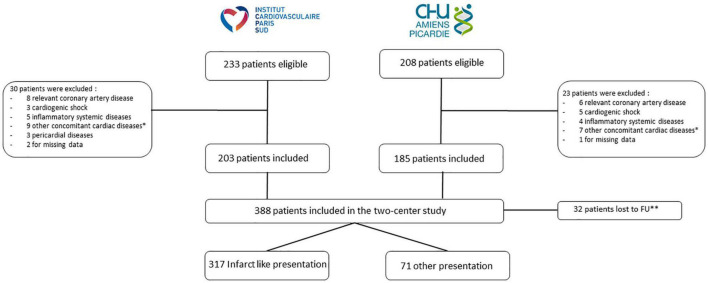
Flow chart of the study. *Other cardiac diseases were chemotherapy-induced cardiomyopathy, valve disease, arrhythmogenic right ventricular dysplasia, hypertrophic cardiomyopathy. ^**^Censored at the date of the last clinical contact. FU, follow-up.

Of the 441 patients who were eligible for this study, 388 were included (Figure 1). Baseline clinical characteristics are reported in Table 1. The mean age was 38.5 years and 77.3% of patients were male. Men were younger with more dilated LV than women (Table 1). Most patients (*N* = 317.82%) presented with chest pain, ECG showing ST segment or T wave abnormalities and a moderate troponin increase. Of these 317 infarct-like patients, 165 (52%) had a rapid coronary angiography for an elevated ST segment in at least 2 contiguous leads, showing angiographically normal arteries. Coronary angiography was waived in the remaining 152 patients (48%) due to the absence of ST elevation and a very low cardiovascular risk profile (mean age 33 years, absence of cardiovascular risk factors). The other symptoms at initial presentation included flu-like syndrome, palpitations, dyspnea and syncope/pre-syncope. Patients with an infarct-like presentation had lower LV EF (*p* = 0.025), more frequent edema (*p* = 0.004) and LGE more frequently involving the subepicardial layer of the myocardium (Table 2). Out of the 388 patients enrolled, 32 (8.2%) were lost to follow-up and were censored at the date of the last clinical contact.

**TABLE 1:** Baseline characteristics of study patients according to gender.

Variables	Total (388)	Men (*n* = 300)	Women (*n* = 88)	*p*-value
Age (years)	38.5 ± 17	35 ± 15	49 ± 18	**<0.001**
History of cardiovascular disease (*N*,%)	6 (1.5)	4 (1.3)	2 (2.3)	0.622
Prior history of myocarditis (*N*,%)	7 (1.8)	7 (2.3)	0 (0.0)	0.358
Recent acute viral infection (*N*,%)	115 (29.7)	96 (32.0)	19 (21.6)	0.064
Chest pain (*N*,%)	317 (81.7)	254 (84.8)	63 (71.6)	0.007
Syncope/pre-syncope (*N*,%)	27 (6.9)	20 (6.7)	7 (8.0)	0.639
Time between symptoms onset and CMR (days)	4 ± 2	4 ± 2	5 ± 4	0.971
Initial LV ejection fraction (%)	56 ± 8	56 ± 8	56 ± 9	0.765
Presence of areas of hypokinesia (*N*,%)	123 (31.7)	91 (30.3)	32 (36.4)	0.299
Initial end-diastolic LV volume (ml/m^2^)	73 ± 19	75 ± 19	67 ± 18	**0.001**
LV dilatation (*N*,%)	37 (9.5)	30 (10.0)	7 (8.0)	0.682
Pericardial effusion (*N*,%)	92 (23.7)	66 (22.0)	26 (29.5)	0.155
***Wall distribution of LGE***				0.206
Subepicardial (*N*,%)	353 (91.0)	276 (92.0)	77 (87.5)	
Midwall (*N*,%)	35 (9.0)	24 (9.0)	11 (12.5)	
Lateral wall involved (*N*,%)	329 (84.8)	256 (85.3)	73 (83.0)	0.613
Presence of T2-hypersignal (*N*,%)	245 (63.1)	197 (65.7)	48 (54.5)	0.061
Myocardial extent of LGE (% myocardial surface area)	10.9 ± 7.4	11.1 ± 7.7	10.6 ± 6.3	0.595

*Continuous variables are expressed as mean ± 1 standard deviation and categorical variables as percentages and counts.*

*CMR, cardiac magnetic resonance; LGE, late gadolinium enhancement; LV, left ventricular.*

*Bold value reffered to p ≤ 0.05.*

**TABLE 2:** CMR characteristics according to initial presentation.

Variables	Infarct-like presentation (*n* = 317)	Other presentations (*n* = 71)	*p*-value
**CMR variables**			
Initial LV ejection fraction (%)	56 ± 7	54 ± 1	**0.025**
LV ejection fraction ≤ 45% (*N*,%)	31 (9.8)	12 (16.9)	0.095
Initial end-diastolic LV volume (ml/m^2^)	74 ± 19	71 ± 18	0.186
LV dilatation (*N*,%)	33 (10.4)	4 (5.6)	0.268
Pericardial effusion (*N*,%)	77 (24.3)	15 (21.1)	0.645
***Wall distribution of LGE***			**0.019**
Subepicardial (*N*,%)	294 (92.2)	59 (83.1)	
Midwall (*N*,%)	23 (7.3)	12 (16.9)	
Lateral wall involved (*N*,%)	267 (84.2)	62 (87.3)	0.587
Septal wall involved (*N*,%)	85 (26.8)	22 (31.0)	0.467
Anterior wall involved (*N*,%)	54 (17.0)	8 (11.3)	0.284
Septal + lateral (*N*,%)	73 (23.0)	21 (29.6)	0.283
Presence of areas of hypokinesia (*N*,%)	102 (32.2)	21 (29.6)	0.778
Presence of LGE in 2 opposed walls (*N*,%)	98 (30.9)	29 (40.8)	0.124
Myocardial extent of LGE (% myocardial surface area)	10.8 ± 7.5	11.7 ± 7.1	0.540
Number of segments with LGE (*N*,%)	4.1 ± 2.4	4.2 ± 2.5	0.696
Presence of T2-hypersignal (*N*,%)	211 (66.6)	34 (47.9)	**0.004**
Number of segments with T2-hypersignal (*N*,%)	2.3 ± 2.3	2.3 ± 1.7	0.968

*Continuous variables are expressed as mean ± 1 standard deviation and categorical variables as percentages and counts.*

*CMR, cardiac magnetic resonance; LGE, late gadolinium enhancement; LV, left ventricular.*

*Bold value reffered to p ≤ 0.05.*

### Cardiovascular Magnetic Resonance Data

**FIGURE 2:**
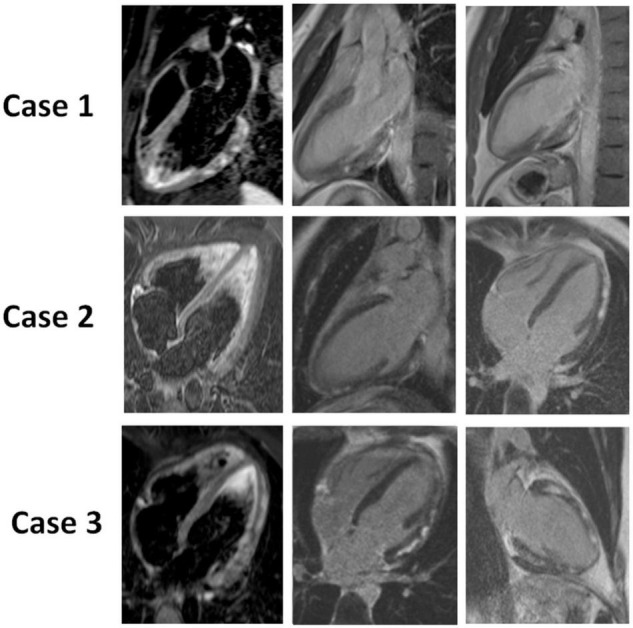
CMR of 3 patients with acute myocarditis: Case 1: a 21-year-old male patient with chest pain and ST elevation. CMR shows a marked focal edema (T2 STIR) and an intramyocardial nodular LGE of the inferolateral wall. Case 2: a 28-year-old male patient with chest pain, dyspnea, ST depression and elevated troponin. CMR shows no focal edema (T2 STIR) and a subepicardial nodular and linear LGE of the latero-apical and infero-apical walls. Case 3: a 33-year-old female patient with chest pain, palpitations, negative T waves and elevated troponin. CMR shows a moderate focal edema (T2 STIR) of the latero-apical wall and a diffuse intramyocardial and subepicardial, nodular and linear LGE. Images in the left panel of the figure are obtained in Black blood T2-weighted short-inversion-time inversion-recovery (STIR) sequence and allow the identification of edema. Images of the mid and right panel are based on inversion-recovery prepared T1-weighted sequences, performed 10 min after intravenous administration of gadolinium chelate and allow the identification of LGE.

The mean time between symptoms onset and CMR performance was 4 ± 2 days. Mean LV EF was 56% and mean LV end-diastolic volume was 73 ml/m2. Initial LV dilatation (LV end-diastolic volume > 100 ml/m^2^) was found in 37 patients (9%). Regional wall motion abnormalities were found in 123 patients (31.7%). Increased signal intensity on T2-STIR images was detected in 245 patients (63.1%). LGE was present in all patients and involved predominantly the lateral wall (84.8%) and subepicardial layer of the LV myocardium (91.0%) (Figure 2). The mean LGE extent was 10.9 ± 7.4% of LV myocardial area. CMR revealed pericardial effusion in 92 patients (23.6%) (Table 1).

### Outcome at the Acute Phase of Acute Myocarditis

Thirty-eight patients (9.8%) experienced an event within 15 days of the AM diagnosis: 1 cardiac death, 22 development or worsening of heart failure (16 were onset and 6 were worsening with mild dyspnea at the time of initial presentation but frank clinical worsening during hospitalization) 13 sustained ventricular arrythmias, and 2 complete atrioventricular blocks. Patients with an early event were older (49 vs. 37 years, *p* < 0.001), had less chest pain but more syncope or pre-syncope at the time of diagnosis than those with no event (both *p* < 0.001) (Table 3). Regarding CMR results, Patients with an early event had lower LV EF (*p* < 0.001), less edema (39.5% vs. 65.7%, *p* = 0.002) but a more extensive LGE (*p* = 0.042) (Table 3). By multivariate logistic regression analysis, the absence of chest pain (OR [95%CI] = 0.35 [0.15–0.82]; *p* = 0.016), the presence of syncope or pre-syncope (OR [95%CI] = 3.56 [1.27–10.02]; *p* = 0.016), lower LV EF (OR [95%CI] = 0.94 [0.91–0.98] per% decrease; *p* = 0.006), myocardial extent of LGE (OR [95%CI] = 1.05 [1.002–1.100] per% increase; *p* = 0.049) and absence of edema (OR [95%CI] = 0.44 [0.19–0.97];*p* = 0.041) were independently associated with occurrence of events in the acute phase of AM.

**TABLE 3:** Patients with/without clinical event at the acute phase of AM.

Variables	Event in the acute phase (*n* = 38)	Absence of event in the acute phase (*n* = 350)	*p*-value
**Clinical variables**			
Age (years)	49 ± 19	37 ± 16	**<0.001**
Male sex (*N*,%)	25 (65.8)	275 (78.6)	0.100
Prior history of myocarditis (*N*,%)	0 (0.0)	7 (2.0)	1.00
Recent acute viral infection (*N*,%)	7 (18.4)	108 (30.9)	0.135
Chest pain (*N*,%)	18 (47.4)	299 (85.4)	**<0.001**
Syncope/pre-syncope (*N*,%)	11 (28.9)	16 (4.6)	**<0.001**
**CMR variables**			
Initial LV ejection fraction (%)	52 ± 13	56 ± 8	**<0.001**
LV ejection fraction ≤ 45% (*N*,%)	13 (34.2)	30 (8.6)	**<0.001**
Initial end-diastolic LV volume (ml/m^2^)	78 ± 21	73 ± 18	0.146
LV dilatation (*N*,%)	6 (15.8)	31 (8.9)	0.237
Pericardial effusion (*N*,%)	10 (26.3)	82 (23.4)	0.690
***Wall distribution of LGE***			0.065
Subepicardial (*N*,%)	31 (81.6)	322 (92.0)	
Midwall (*N*,%)	7 (18.4)	28 (8.0)	
Lateral wall involved (*N*,%)	35 (92.1)	294 (84.0)	0.318
Septal wall involved (*N*,%)	13 (34.2)	94 (26.9)	0.343
Anterior wall involved (*N*,%)	5 (5.3)	60 (17.1)	0.063
Septal + lateral (*N*,%)	12 (31.6)	82 (23.4)	0.318
Presence of areas of hypokinesia (*N*,%)	17 (44.7)	106 (30.3)	0.097
Presence of LGE in 2 opposed walls (*N*,%)	15 (39.5)	112 (32.0)	0.366
Myocardial extent of LGE (% myocardial surface area)	13.4 ± 8.6	10.6 ± 7.0	**0.042**
Number of segments with LGE (*N*,%)	4.5 ± 2.8	4.0 ± 2.4	0.311
Presence of T2-hypersignal (*N*,%)	15 (39.5)	230 (65.7)	**0.002**
Number of segments with T2-hypersignal (*N*,%)	2.6 ± 2.5	2.3 ± 2.2	0.184

*Continuous variables are expressed as mean ± 1 standard deviation and categorical variables as percentages and counts.*

*AM, acute myocarditis; CMR, cardiac magnetic resonance; LGE, late gadolinium enhancement, LV, left ventricular.*

*Bold value reffered to p ≤ 0.05.*

### Major Adverse Cardiac Events During Follow-Up

**FIGURE 3:**
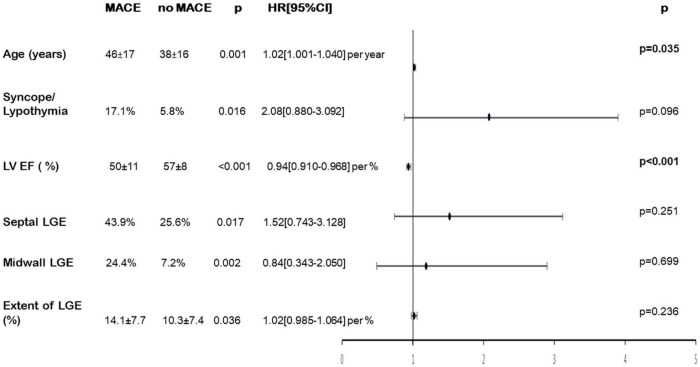
Univariate and multivariate analysis of variables associated with MACE during follow-up. Forrest plot: Hazard ratio and 95% confidence interval for risk of MACE during follow-up. LGE, late gadolinium enhancement; LV EF, left ventricular ejection fraction; MACE, major cardiac events.

Forty-one patients (10.5%) developed at least one MACE during follow-up [median: 7.5(IQR:6.6–8.9) years] including cardiac death/heart transplantation in 6 patients, documented sustained ventricular tachycardia in 6 patients, hospitalization for cardiac causes in 15 patients and hospitalization for heart failure in 14 patients. Patients who developed MACE during follow-up were older (46 vs. 38 years, *p* = 0.001) and had experienced more syncope/pre-syncope during the acute episode (*p* = 0.016) (Table 4). Patients with MACEs had lower LV EF (*p* < 0.001), a more extensive LGE (*p* = 0.036) which involved more frequently the septal wall (*p* = 0.017), and was more often localized in the midwall layer of the LV (*p* = 0.002) compared to patients without MACEs. By multivariate Cox analysis, only age (HR [95%CI] = 1.021 [1.001–1.041] per year; *p* = 0.035), and lower LV EF (HR [95%CI] = 0.94 [0.91–0.97] per% decrease; *p* < 0.001), remained independently associated with MACE occurrence (Figure 3). Only lower LV EF (HR [95%CI] = 0.90 [0.87–0.94] per% decrease; *p* < 0.001), remained independently associated with the occurrence of MACE in patients presenting with edema and LGE (*n* = 245).

**TABLE 4:** Patients with/without MACE at follow-up.

Variables	MACE at follow-up (*n* = 41)	Absence of MACE at follow-up (*n* = 347)	*p*-value
**Clinical variables**			
Age (years)	46 ± 17	38 ± 16	**0.001**
Male sex (*N*,%)	30 (73.2)	270 (77.8)	0.554
Prior history of myocarditis (*N*,%)	1 (2.4)	6 (1.7)	0.545
Recent acute viral infection (*N*,%)	11 (26.8)	104 (30.0)	0.599
Chest pain (*N*,%)	29 (70.7)	288 (83.0)	0.084
Syncope/pre-syncope (*N*,%)	7 (17.1)	20 (5.8)	**0.016**
**CMR variables**			
Initial LV ejection fraction (%)	50 ± 11	57 ± 8	**<0.001**
LV ejection fraction ≤ 45% (*N*,%)	14 (34.1)	29 (8.4)	**<0.001**
Initial end-diastolic LV volume (ml/m^2^)	76 ± 24	73 ± 18	0.241
LV dilatation (*N*,%)	7 (15.9)	30 (8.7)	0.092
Pericardial effusion (*N*,%)	9 (22.0)	83 (23.9)	0.849
***Wall distribution of LGE***			**0.002**
Subepicardial (*N*,%)	31 (75.6)	322 (92.8)	
Midwall (*N*,%)	10 (24.4)	25 (7.2)	
Lateral wall involved (*N*,%)	36 (87.8)	293 (84.4)	0.818
Septal wall involved (*N*,%)	18 (43.9)	89 (25.6)	**0.017**
Anterior wall involved (*N*,%)	5 (12.2)	57 (16.4)	0.653
Septal + lateral (*N*,%)	16 (39.0)	78 (22.5)	**0.032**
Presence of areas of hypokinesia (*N*,%)	18 (43.9)	105 (30.3)	0.110
Presence of LGE in 2 opposed walls (*N*,%)	18 (43.9)	109 (31.4)	0.115
Myocardial extent of LGE (% myocardial surface area)	14.1 ± 7.7	10.3 ± 7.4	**0.036**
Number of segments with LGE (*N*,%)	5.1 ± 2.8	3.9 ± 2.3	**0.004**
Presence of T2-hypersignal (*N*,%)	21 (51.2)	224 (64.6)	0.123
Number of segments with T2-hypersignal (*N*,%)	2.8 ± 2.7	2.2 ± 2.1	0.100

*Continuous variables are expressed as mean ± 1 standard deviation and categorical variables as percentages and counts.*

*CMR, cardiac magnetic resonance; LGE, late gadolinium enhancement; LV, left ventricular; MACE, major cardiac events.*

*Bold value reffered to p ≤ 0.05.*

**FIGURE 4:**
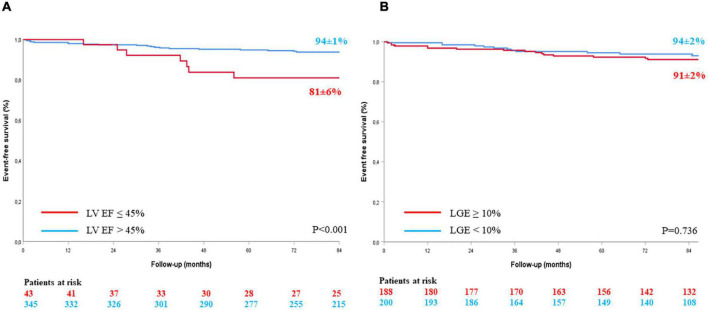
Kaplan Meier event-free survival curves according to LV EF **(A)** and LGE extension **(B)**. LGE, late gadolinium enhancement; LV EF, left ventricular ejection fraction.

Estimated 7-year event free survival was 94 ± 1% for patients with LV EF > 45% and 81 ± 6% for patients with LV EF ≤ 45% (Log Rank *p* < 0.001) (Figure 4A). Estimated 7-year event free survival was 94 ± 2% for patients with LGE extent < 10% of myocardial mass and 91 ± 2% for patients with LGE extent ≥ 10% of myocardial mass (Log Rank *p* = 0.736) (Figure 4B).

### Recurrence of Acute Myocarditis

During follow-up, 30 patients (7.7%) experienced a recurrence of AM with a mean delay of 4.9 ± 3.1 years after the initial episode. Patients with recurrent myocarditis reported more viral syndrome at the time of the index episode (50% vs. 27.9%; *p* = 0.020) and had more often a prior history of myocarditis before this index episode (*p* = 0.012) (Table 5). By multivariate Cox analysis, these two variables remained independently associated with an AM recurrence during follow-up (HR [95%CI] = 5.74 [1.72–19.22]; *p* = 0.005 for prior myocarditis and HR [95%CI] = 4.21 [1.91–9.28]; *p* < 0.001 for viral syndrome).

**TABLE 5:** Patients with/without recurrence of AM during follow-up.

Variables	Recurrence of AM during follow-up (*n* = 30)	No recurrence of AM during follow-up (*n* = 358)	*p*-value
**Clinical variables**			
Age (years)	46 ± 17	38 ± 16	0.202
Male sex (*N*,%)	24 (80.0)	276 (77.1)	0.824
Prior history of myocarditis (*N*,%)	3 (10.0)	4 (1.1)	**0.012**
Recent acute viral infection (*N*,%)	15 (50.0)	100 (27.9)	**0.020**
Chest pain (*N*,%)	26 (86.7)	291 (81.3)	0.625
Syncope/pre-syncope (*N*,%)	2 (6.7)	25 (7.0)	1.00
**CMR variables**			
Initial LV ejection fraction (%)	50 ± 11	57 ± 8	0.477
LV ejection fraction ≤ 45% (*N*,%)	4 (13.3)	29 (8.4)	0.760
Initial end-diastolic LV volume (ml/m^2^)	76 ± 24	73 ± 18	0.461
LV dilatation (*N*,%)	4 (13.3)	33 (9.2)	0.512
Pericardial effusion (*N*,%)	6 (20.0)	86 (24.0)	0.823
***Wall distribution of LGE***			0.744
Subepicardial (*N*,%)	27 (90.0)	326 (91.1)	
Midwall (*N*,%)	3 (10.0)	31 (8.9)	
Lateral wall involved (*N*,%)	24 (80.0)	305 (85.2)	0.430
Septal wall involved (*N*,%)	10 (33.3)	97 (27.1)	0.824
Anterior wall involved (*N*,%)	8 (26.7)	54 (15.1)	0.117
Septal + lateral (*N*,%)	8 (26.7)	86 (24.0)	0.824
Presence of areas of hypokinesia (*N*,%)	13 (43.3)	110 (30.7)	0.158
Presence of LGE in 2 opposed walls (*N*,%)	9 (30.0)	118 (33.0)	0.841
Myocardial extent of LGE (% myocardial surface area)	14.1 ± 7.7	10.3 ± 7.4	0.609
Number of segments with LGE (*N*,%)	5.1 ± 2.8	3.9 ± 2.3	0.303
Presence of T2-hypersignal (*N*,%)	21 (70.0)	224 (62.6)	0.555
Number of segments with T2-hypersignal (*N*,%)	2.8 ± 2.7	2.2 ± 2.1	0.100

*Recurrence of AM was defined as a new episode of clinically suspected AM, at least 6 months after the index episode, with the presence of edema in T2 STIR and LGE.*

*AM, acute myocarditis; CMR, cardiac magnetic resonance; LGE, late gadolinium enhancement; LV, left ventricular.*

*Bold value reffered to p ≤ 0.05.*

## Discussion

**FIGURE 5:**
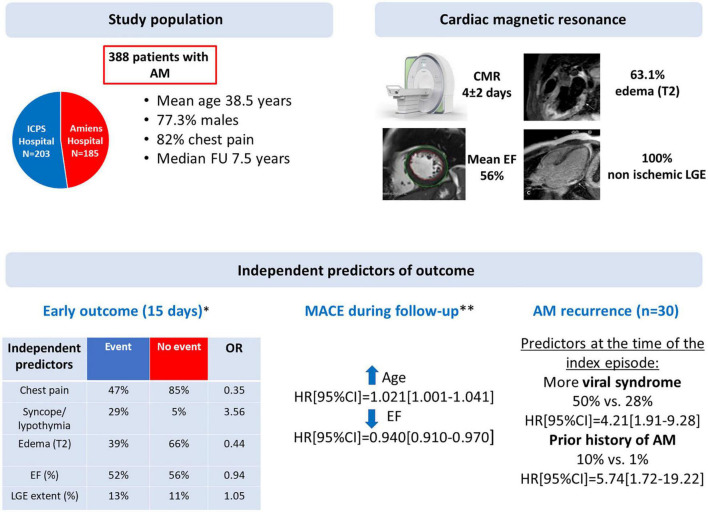
Summary of the study: study population, CMR characteristics and independent predictors of outcome. AM, acute myocarditis; AV, atrioventricular; CI, confidence interval; CMR, cardiac magnetic resonance; EF, ejection fraction; HR, hazard ratio; LGE, late gadolinium enhancement; MACE, major cardiac events; OR, odd ratio. *Cardiac death, heart failure, ventricular arrhythmia or complete AV block (*n* = 38). **Cardiac death, cardiac transplantation, sustained ventricular tachycardia hospitalization for heart failure or for cardiac causes (*n* = 41).

In this two-center study of 388 consecutive patients with a diagnosis of AM based on CMR with a mild presentation, the absence of edema, reduced LV EF, and extent of LGE were associate with early outcome while the only independent CMR predictor of an adverse long-term outcome was an initial impairment of LV EF. The presence and extent of acute focal myocardial edema and the extent of myocardial tissue damage on LGE-CMR were not independently related to clinical outcome over a long median follow-up of 7.5(6.6–8.9) years (Figure 5).

Interestingly, the presence of edema was associated with a better early outcome, suggesting a potential protective role of inflammatory response in the acute phase of AM. McLellan et al. showed consistent results in patients presenting with acute cardiomyopathy, demonstrating that the extent of myocardial inflammation identified by high global relative enhancement was predictive of left ventricular function recovery (21). Vermes et al. reported in a population of 37 AM, that the presence of global and/or regional edema on admission was the sole independent predictor of a subsequent recovery of LV EF, likely reflecting a recovery of reversibly injured myocardium (22). Our data do not explain the mechanism by which edema appears protective. However, as in AM, interstitial space could be increased by the presence of edema and not just by focal fibrosis, we can postulate, that in patients with both LGE and edema, there is active inflammation with the possibility of future healing, whereas in those without edema, LGE would correspond to fibrosis that would be more likely to persist over time. However, it is noteworthy that identification of edema is not always easy with T2 STIR sequences because of several limitations. Indeed, the high signal from stagnant blood in the LV cavity, the low signal-to-noise ratio, and the high sensitivity to myocardial motion complicates its identification. More recent sequences with quantitative T1 and T2 mapping techniques might be well suited to detect myocardial edema or other myocardial tissue alterations in AM with higher sensitivity, but they were not available at the time of our study (23). Nevertheless, these sequences also present some limitations. Firstly, the values can vary significantly between CMR machines and from one manufacturer to another. Secondly, thresholds for the diagnosis of AM are currently missing and comparative data with histopathology is lacking. It also seems difficult in clinical practice to make the diagnosis of AM using only T1/T2 mapping in patients without focal edema and without LGE, with the risk of over diagnosing this condition.

In agreement with previous studies of patients with AM diagnosed either by EMB or CMR (4, 18, 24–26), the initial LV EF determined by CMR was an independent predictor of early and long-term outcome. Patients with AM and LGE are more likely to have greater myocardial damage than patients with AM but no LGE as it may favor progressive left ventricular remodeling and dysfunction and eventually leads to heart failure (14, 26). Furthermore, LGE may play a key role in the genesis of ventricular arrhythmias by promoting reentrant circuits (27). According to a recent meta-analysis, the presence of LGE on baseline CMR in AM is an important independent prognostic marker that portends an increased risk of major adverse cardiac events (14). However, studies evaluated the impact of LGE presence in AM are very heterogeneous. Grün et al. reported that the presence of LGE was independently predictive of long-term all-cause and cardiac mortality in patients with myocarditis proven by biopsy (15). It is important to note that our study population was very different from the latter study where a substantial number of patients had severe heart failure and LV dysfunction (45% in NYHA III-IV, mean LVEF: 45%). Viral genomes are frequently detected in the EMBs of patients with left ventricular systolic dysfunction and the persistence of these viruses, often in the form of recurrent infections, may be involved in the transition from myocarditis to dilated cardiomyopathy (28), or may precipitate the development of heart failure (29). In our study, the presence of LGE was mandatory to establish the diagnosis of AM and therefore, we could not assess the impact of its presence on the prognosis.

The prognostic impact of LGE extent in AM is debated. Gräni et al. found that a 10% increase in the extent of LGE led to a 79% increase in the risk of MACE, after adjustment for LVEF (17). However, their population was different from ours. Indeed, unlike in the current study, only 44% of patients had LGE but almost 50% of patients had heart failure and/or left ventricular dysfunction (mean LVEF 49% vs. 56%) which may partly explain the conflicting results (17). White et al. have recently reported in a series of 100 patients with AM diagnosed by CMR, that neither the presence nor the extent of LGE was associated with improvement in LV EF or reduction in LV end-diastolic volume index at 12 months (19). A pilot study by Aquaro et al. involving patients with AM, preserved LV EF and NYHA class I, suggested that LGE in this acute setting is not synonymous with definitive fibrosis and that the persistence of LGE on repeat CMR at 6 months is associated with poor outcome (30). In our study, the extent of LGE was independently associated with early outcome but not with long-term MACE after adjustment to LV EF, which remained the strongest prognostic marker. It is therefore possible that the extent of LGE at initial presentation reflects the severity of the disease in the early phase, but not at long term, as its course is not predictable and only its persistence or worse its progression may be associated with long term events.

A recent study suggested a relationship between anteroseptal localization of LGE and worse prognosis (16). whereas in the current study, septal localization of LGE was associated with MACE in univariate analysis but not in multivariate analysis. However, the prevalence of anteroseptal involvement was lower in our study (39% of patients with LGE vs. 27% in our study), which may explain this discrepancy. In concordance with our data, Aquaro et al. reported that the extent of LGE was not associated with MACE occurrence (16).

It is important to note that the current findings are only valid in patients with a diagnosis of AM based on CMR with a predominantly infarct-like presentation and without hemodynamic compromise. However, this is the most common presentation of the disease in clinical practice by far. The diagnosis of AM was based on a combination of clinical, electrocardiographic and/or biological arguments, as well as CMR, which showed intramyocardial and/or subepicardial lesions strongly indicative of acute non-ischemic tissue damage. The CMR diagnostic criteria were slightly different from the original Lake-Louise criteria (in effect at the time of the study) according to which a diagnosis of AM is made when at least 2 parameters among T2 hypersignal, early enhancement and late enhancement are met (12). In our study, the presence of subepicardial and/or intramyocardial LGE lesions was required for the diagnosis as this parameter is more specific and limits the possibility of including patients with myocardial edema due to another cause. Similarly, we did not include clinical suspicions of AM with a negative CMR, because we believe that the diagnosis may be questionable in those situations. Finally, to our knowledge, we report the largest study of patients with acute myocarditis confirmed by the presence of non-ischemic typical myocarditis LGE patterns on CMR with the longest follow-up (median 7.5 years).

### Limitations

Our study presents the limitations inherent to retrospective analyses. Coronary artery disease has not been ruled out by specific examination in all patients. However, none of the patients had an LGE pattern consistent with myocardial infarction, and coronary angiography was performed when deemed necessary to rule out an acute coronary syndrome. EMB was not performed to confirm the diagnosis of AM. This study did not provide comparisons between CMR lesions and biomarkers to predict clinical outcomes because these markers were not available for all patients. Also, the empirical treatment based on β-blockers and angiotensin-converting enzyme inhibitors might have influence the outcome of patients. T1 and T2 mapping techniques were not available at the time of our study and we cannot rule out the possibility that patients without focal edema might have another diagnosis, even if all patients had a clinical presentation suggestive of AM. CMR does not allow to distinguish the most common lymphocytic-dominant myocarditis from more severe forms of AM such as giant cell, eosinophilic and sarcoid myocarditis, which might be associated with poor outcomes. However, our population consisted only of hemodynamically stable patients with AM of presumed viral origin. Recent data suggest that patients with inherited cardiomyopathies can present with acute myocarditis and that they tend to have worse prognosis compared to “pure” myocarditis (31-33). Unfortunately, this interesting information was not known during the inclusion period of the study and therefore data on family history of cardiomyopathy or AM and genetic testing were not available in our database. Although follow-up is long, it is possible that some events occurred later and that LGE extent could be associated with those later events. Finally, all patients included in this study had early CMR, which represents a selection bias because it excluded the most severe patients.

## Conclusion

In consecutive routine patients diagnosed with acute myocarditis by CMR with mild presentation and without severe hemodynamic compromise, the absence of edema, reduced LV EF, and extent of LGE were associated with early outcome. In contrast, only age and initial LV EF impairment were predictors of long-term adverse outcome, whereas the presence of myocardial edema and the extent of LGE were not.

## Data Availability Statement

The raw data supporting the conclusions of this article will be made available by the authors, without undue reservation.

## Ethics Statement

The studies involving human participants were reviewed and approved by the Ethics committee of ICPS and Amiens University Hospital. Written informed consent for participation was not required for this study in accordance with the national legislation and the institutional requirements.

## Author Contributions

JG, FS, and YB: design of the study. FS, CTa, CD, WB, IL, and YB: data acquisition. YB and FS: statistical analysis. JG, YB, and FS: analysis and interpretation of the data, drafting of the manuscript. All authors substantially contributed to the manuscript and reviewed the manuscript.

## Conflict of Interest

The authors declare that the research was conducted in the absence of any commercial or financial relationships that could be construed as a potential conflict of interest.

## Publisher’s Note

All claims expressed in this article are solely those of the authors and do not necessarily represent those of their affiliated organizations, or those of the publisher, the editors and the reviewers. Any product that may be evaluated in this article, or claim that may be made by its manufacturer, is not guaranteed or endorsed by the publisher.
